# Exploring Potential Medications for Alzheimer’s Disease with Psychosis by Integrating Drug Target Information into Deep Learning Models: A Data-Driven Approach

**DOI:** 10.3390/ijms26041617

**Published:** 2025-02-14

**Authors:** Oshin Miranda, Chen Jiang, Xiguang Qi, Julia Kofler, Robert A. Sweet, Lirong Wang

**Affiliations:** 1 Computational Chemical Genomics Screening Center, Department of Pharmaceutical Sciences, School of Pharmacy, University of Pittsburgh, Pittsburgh, PA 15213, USA; osm7@pitt.edu (O.M.); chj51@pitt.edu (C.J.); xiq24@pitt.edu (X.Q.); 2 Division of Neuropathology, Department of Pathology, University of Pittsburgh, Pittsburgh, PA 15213, USA; koflerjk@upmc.edu; 3 Alzheimer Disease Research Center, University of Pittsburgh, Pittsburgh, PA 15213, USA; sweetra@upmc.edu; 4 Department of Psychiatry, School of Medicine, University of Pittsburgh, Pittsburgh, PA 15213, USA

**Keywords:** Alzheimer’s disease, psychosis, system pharmacology, drug target, artificial intelligence, biomarker identification

## Abstract

Approximately 50% of Alzheimer’s disease (AD) patients develop psychotic symptoms, leading to a subtype known as psychosis in AD (AD + P), which is associated with accelerated cognitive decline compared to AD without psychosis. Currently, no FDA-approved medication specifically addresses AD + P. This study aims to improve psychosis predictions and identify potential therapeutic agents using the DeepBiomarker deep learning model by incorporating drug–target interactions. Electronic health records from the University of Pittsburgh Medical Center were analyzed to predict psychosis within three months of AD diagnosis. AD + P patients were classified as those with either a formal psychosis diagnosis or antipsychotic prescriptions post-AD diagnosis. Two approaches were employed as follows: (1) a drug-focused method using individual medications and (2) a target-focused method pooling medications by shared targets. The updated DeepBiomarker model achieved an area under the receiver operating curve (AUROC) above 0.90 for psychosis prediction. A drug-focused analysis identified gabapentin, amlodipine, levothyroxine, and others as potentially beneficial. A target-focused analysis highlighted significant proteins, including integrins, calcium channels, and tyrosine hydroxylase, confirming several medications linked to these targets. Integrating drug–target information into predictive models improves the identification of medications for AD + P risk reduction, offering a promising strategy for therapeutic development.

## 1. Introduction

Alzheimer’s disease (AD) is a neurodegenerative condition affecting millions of Americans. Around 50% of AD patients develop psychotic symptoms, a condition referred to as AD with psychosis (AD + P), which is characterized by hallucinations and delusions [1]. These symptoms typically manifest during the early to moderate stages of cognitive decline. AD + P patients exhibit more severe cognitive decline, higher rates of agitation, depression, and functional impairment compared to AD without psychosis (AD-P) [2]. Addressing these psychotic manifestations poses a significant challenge in dementia management, warranting urgent attention and effective therapeutic interventions.

Recent advancements have highlighted genomic, neuroimaging, and neurobiological findings of psychosis in AD, offering crucial mechanistic insights. Genomic studies have revealed significant heritability estimates for AD psychosis, identifying key risk loci in genes like *ENPP6* and *SUMF1* [3]. Notably, a modest association with the apolipoprotein E (APOE) ε4 allele has been observed, influencing diagnostic considerations. Moreover, genetic correlation analyses suggest shared genetic liabilities between AD psychosis and other psychiatric conditions [4]. Epigenomic investigations have unveiled hypomethylation patterns in genes implicated in AD psychosis, overlapping with variants linked to schizophrenia [5]. Neuroimaging studies have revealed structural and functional alterations in brain regions linked to Alzheimer’s disease psychosis, with a notable emphasis on changes in the right hemisphere. Post mortem analyses highlight the role of neurofibrillary tangle pathology and synaptic disruption in AD psychosis, implicating tau hyperphosphorylation and neurotransmission dysregulation [1]. Comorbidities like Lewy body pathology and vascular pathology contribute to the multifactorial etiology of AD psychosis [6]. Overall, these findings offer significant insights into the mechanisms driving psychosis in AD [7,8].

Non-pharmacological management approaches are often prioritized as the initial interventions for addressing the behavioral and psychological symptoms of dementia (BPSD), including psychosis, due to their established safety and effectiveness [9]. Approaches include avoiding confrontation, acknowledging and addressing the underlying emotions driving delusions, and assessing whether psychotic symptoms are distressing before considering pharmacological interventions. Pharmacological interventions, historically relying on antipsychotic medications, pose significant risks and adverse effects in dementia patients. While some atypical antipsychotics, like aripiprazole and risperidone, have demonstrated modest efficacy, their use is associated with adverse effects and mortality risks [10]. Other agents, including citalopram and sertraline, have shown efficacy in treating agitation in AD, with potential benefits for psychotic symptoms [11,12]. Cholinesterase inhibitors, commonly used for cognitive symptoms in AD, may also have a role in managing psychosis, although further studies are needed [13]. Additionally, growing evidence points to a possible connection between vitamin D deficiency and the occurrence of AD psychosis [14], warranting further investigation. Overall, while non-pharmacological interventions remain the preferred approach for managing psychosis in dementia, ongoing research into pharmacological options offers hope for safer and more effective treatments in the future.

Psychosis in Alzheimer’s disease (AD + P) has a significant genetic component, with studies revealing strong familial clustering and heritability rates of up to 61% [15]. Research has focused on genome-wide association studies (GWASs), candidate gene associations, and copy number variations (CNVs) to find genetic risk factors for AD + P. However, these studies have produced incomplete results, highlighting the need for replication in larger cohorts to validate these associations and deepen our understanding of the genetic underpinnings of AD + P [16,17]. Understanding the genetic basis of AD + P remains crucial for developing targeted therapeutic interventions aimed at alleviating the disease burden.

Electronic medical records (EMRs) play an essential role in clinical documentation and practice, providing valuable information for the study of disease progression. Despite their potential, limited research has thoroughly examined the predictive potential of multimodal data extracted from EMRs, such as medication use, diagnoses, and laboratory test results, in identifying high-risk patient outcomes [18,19,20]. This study aims to use EMR data to predict AD risks and identify drug targets and evidence-based interventions. By incorporating biomarkers, demographics, comorbidities, medication use, drug targets, and other characteristics, our approach facilitates the creation of tailored treatment plans and explores alternative options when conventional treatments prove ineffective. While AD + P remains a significant public health concern, our ongoing research aims to provide a targeted approach to manage this syndrome effectively.

Deep learning or data mining algorithms are effective tools for analyzing large-scale EMR data [21]. These algorithms enable the identification of hidden patterns and relationships within the data, uncovering significant insights that might have otherwise been overlooked [22]. Recent advancements in artificial intelligence (AI) and deep learning have revolutionized biomedical research, particularly in understanding and treating complex diseases like Alzheimer’s disease (AD). AI-driven frameworks have demonstrated remarkable potential in integrating multimodal datasets to uncover pathobiology and therapeutic opportunities. For instance, Xu et al. developed a network topology-based deep learning framework (NETTAG) that combined GWAS data and multi-omics data to identify druggable targets and repurpose existing medications, successfully linking drugs like ibuprofen and gemfibrozil to reduced AD incidence [23]. Similarly, Kale et al. highlighted how AI enhances early AD diagnosis through neuroimaging and cognitive assessments and facilitates personalized treatment through drug repurposing and prognostic modeling [24]. Furthermore, Maudsley et al. emphasized the critical role of informatics and deep learning in deconstructing Big Data for drug discovery and integrating diverse datasets, such as transcriptomics, proteomics, and metabolomics, for actionable insights [25]. While these studies demonstrate the feasibility and utility of deep learning in AD research, they focus predominantly on AD without addressing its psychosis-associated subtype, AD + P, which presents unique clinical and pathological challenges. In this study, our primary focus is on the identification of drug targets using advanced deep learning techniques applied to EMR data. By integrating protein target information into our updated DeepBiomarker model, we aim to uncover hidden relationships between medications and their potential to impact the progression of AD + P. The refined model is tailored to high-risk cohorts, such as AD + P, which allows for the identification of new medications that may prevent psychosis. As an additional enhancement, we expanded our inclusion criteria to account for the underdiagnosis of AD + P in clinical practice by incorporating patients who used antipsychotics after an AD diagnosis. This adjustment strengthens the model’s capability to capture relevant cases, further improving the robustness of our findings.

## 2. Results

### 2.1. The Cohort Information and Model Performance of Updated DeepBiomarker on the Psychosis Prediction

We identified 16,294 patients diagnosed with AD. Among them, we further categorized 2861 cases as patients who developed AD + psychosis (AD + P) within three months after their index dates, while 9799 controls did not develop AD + P within the same timeframe. The index date for each patient was defined as the date of any clinical encounter occurring after the AD diagnosis but before the diagnosis of AD + P. All patients had more than one year of EMR data available before their index dates. The mean age of AD + P patients was 80.8 years (standard deviation: 8.34), compared to 83.4 years (standard deviation: 8.16) in the control group. In terms of gender distribution, the case cohort comprised 1830 females and 1031 males, whereas the control cohort had 6452 females and 3347 males.

The dataset was split into training, validation, and testing subsets in an 8:1:1 ratio. The validation datasets were utilized for optimizing parameters in the updated DeepBiomarker model, while the testing set was used to assess its performance. The predictive accuracy of the updated DeepBiomarker model is presented in Table 1, where a higher area under the curve (AUC) indicates greater accuracy in predictions. Notably, both time-aware long short-term memory (TLSTM) and the reverse time attention model (RETAIN) in the updated DeepBiomarker model achieved AUCs above 0.90 in both the validation and test sets, demonstrating a high predictive accuracy. In comparison, logistic regression showed lower accuracy, with AUCs of 0.797 and 0.789 in the validation and test datasets, respectively.

To further understand the contributions of individual feature types to predictive performance, we conducted ablation experiments using the RETAIN model. As shown in Table 1, the removal of laboratory results led to a slight decrease in Test AUC (from 0.915 to 0.911), while excluding medication information resulted in a Test AUC of 0.905. The most substantial impact was observed when the diagnosis information was removed, with the Test AUC dropping to 0.858. Similarly, the F1 score followed a similar trend, with values decreasing more notably when the diagnosis information was excluded (from 0.872 to 0.789), reinforcing the pivotal role of diagnosis in prediction accuracy. These results highlight the critical importance of diagnosis information in predicting psychosis risk among AD patients, followed by medication information and laboratory results.

### 2.2. Important Biomarkers for Psychosis Prediction

#### 2.2.1. Method 1: Drug-Focused Analysis

Method 1 (drug-focused analysis) uses a perturbation-based importance analysis to evaluate the impact of drugs by replacing them with their corresponding targets, grouped by protein IDs. The relative contribution (RC) values for these targets are calculated to identify beneficial medications that may have an impact on AD + P, with detailed results shown in Table 2. An RC value greater than 1 indicates a higher association with cases compared to the controls, suggesting a potential risk factor, while an RC value below 1 denotes a protective effect. These findings emphasize the critical features identified using Method 1.

Table 2 summarizes several medications with RC values below 1, indicating they are associated with reduced rates of AD + P onsets. Based on their RC values, medications with RC values greater than 1 are categorized as high risk, while those with RC values less than 1 are considered low risk or beneficial options for treatment. High-risk medications include ketoconazole (1.96), which treats fungal infections and some endocrine disorders [26]. Low-risk/beneficial medications include donepezil (0.88) [27] and memantine (0.89) [28] for Alzheimer’s disease; levothyroxine (0.78) [29] for hypothyroidism; metoprolol (0.80) [30] and atenolol (0.76) [31] for high blood pressure and heart conditions; furosemide (0.83) [32] for fluid retention; lisinopril (0.84) [33] for hypertension and heart failure; amlodipine (0.82) [34] for hypertension and angina; vitamin D3 (0.90) [35] for bone health; simvastatin (0.82) [36] and rosuvastatin (0.68) [37] for cholesterol management; pantoprazole (0.72) [38] for acid-related conditions; sulfamethoxazole (0.60) and trimethoprim (0.66) [39] as antibiotics for infections; rivastigmine (0.67) [40] for dementia; ibuprofen (0.67) [41] for pain and inflammation; glimepiride (0.39) [42] for diabetes management; esomeprazole (0.59) [43] for acid-related issues; irbesartan (0.39) [44] for high blood pressure and kidney protection; methenamine (0.45) [45] to prevent UTIs; fludrocortisone (0.37) [46] for adrenal insufficiency; phenazopyridine (0.29) [47] for alleviating urinary tract discomfort; and oxybutynin (0.52) [48] for overactive bladder. This categorization helps in assessing the relative risks and benefits of these medications in our study.

#### 2.2.2. Method 2: Target-Focused Analysis

Method 2 (target-focused analysis) emphasizes the drug targets rather than the drugs themselves. This method involves pooling drugs based on shared targets, which can increase sample sizes and enhance statistical power. By focusing on drug targets as the primary inputs, this approach offers a deeper understanding of drug–target interactions and their implications, which is key. In Table 3, we have listed the feature name, the protein name associated with the feature name (i.e., drug target), and their associated drugs and IDs. Method 2 enables the development of targeted treatment and prevention strategies, potentially improving patient outcomes. This method is different from Method 1, as it provides a more detailed and actionable understanding of the interactions between drugs and their targets, which is essential for developing precise therapeutic interventions for a high-risk population such as AD + P. Additionally, several common comorbid diseases among AD patients identified by perturbation-based contribution analysis is shown in Supplementary Table S1.

## 3. Discussion

Certain drugs were identified in our drug-focused (Method 1) or target-focused (Method 2) analyses. Although a drug may have multiple targets, not all of these targets are significant. Additionally, interactions with other drugs, though rare, can also influence these findings. To dive deeper into the identification of specific biomarkers specific to AD + P, we categorized our top medications, validated through previous research endeavors and current findings.

### 3.1. Impact of Feature Types: Insights from Ablation Experiments

To better understand the contribution of different data types to the predictive performance of our model, we conducted ablation experiments using the RETAIN model by systematically removing lab results, medication information, and diagnosis information. Our findings revealed that the removal of diagnosis information resulted in the most significant drop in performance metrics (Test AUC decreased to 0.858), underscoring its pivotal role in predicting psychosis risk among AD patients. Diagnosis codes likely capture critical clinical insights, such as comorbid conditions and psychotic symptoms, that directly inform risk prediction.

Medication information also demonstrated substantial importance, with a notable decline in Test AUC (0.905) and F1 score upon its removal. This suggests that drug–target interactions, as reflected by medication features, play a meaningful role in assessing psychosis risk and identifying potential therapeutic candidates. Lab results, while contributing to prediction accuracy, had a relatively smaller impact compared to diagnosis and medication information.

These results not only validate the inclusion of multimodal information in our model but also highlight the value of clinical data integration in improving the predictive accuracy for complex conditions like AD + P.

### 3.2. Effect of Medication Use on AD + P Prediction

**Figure 1:**
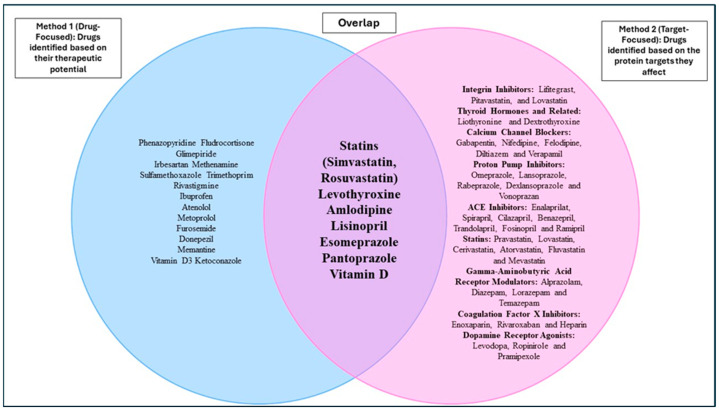
Overlap of potential drug options for AD + P identified by Method 1 and Method 2.

In this section, we focus on the overlapping drugs (shown in Figure 1) identified through both Method 1 (drug-focused) and Method 2 (target-focused) approaches. Statins (simvastatin and rosuvastatin), levothyroxine, amlodipine, lisinopril, and vitamin D were predicted to have beneficial effects. These overlapping medications represent potential therapeutic candidates for AD + P, as they are supported by evidence from both approaches. By confirming the findings from Method 1 using the target-specific results from Method 2, we can further strengthen our understanding of these drugs’ roles in AD + P treatments.

Esomeprazole and pantoprazole have emerged as potential candidates when comparing the overlap between Method 1 and Method 2. Proton pump inhibitors (PPIs), such as esomeprazole and pantoprazole, have been investigated for their potential impact on neurological health. Booker et al. reported in a case–control study in which a statistically significant reduction in dementia risk was associated with PPI use [49]. Goldstein et al. performed a longitudinal study on over 10,000 participants and found that regular or intermittent PPI use was not associated with an increased risk of dementia or Alzheimer’s disease [50]. However, Haenisch et al. and Gomm et al. suggest an increased risk of dementia associated with long-term PPI use [51,52]. Mechanistic studies further suggest that certain PPIs may influence the amyloid beta (Aβ) metabolism. Badiola et al. demonstrated in animal models that lansoprazole, a PPI similar to esomeprazole, increased Aβ production and altered β- and γ-secretase activity [53]. Although these findings do not directly implicate esomeprazole or pantoprazole, they underscore the need for caution when repurposing PPIs for neuropsychiatric conditions, like AD + P. The mixed evidence surrounding esomeprazole and pantoprazole’s impacts on cognitive health highlights the importance of balancing their potential benefits against the risks when considering their repurposing for AD + P.

Gabapentin has shown effects in managing behavioral symptoms in dementia. Moretti et al. reported in 2001 that gabapentin reduced anxiety, aggressiveness, and caregiver stress in patients with AD exhibiting behavioral disturbances [54]. Similarly, his open-label case series of 20 patients in 2003 also concluded that gabapentin could benefit the patients from reductions in agitation, anxiety, and aggressiveness [55]. However, concerns exist regarding its cognitive effects. Huang et al. identified an increased risk of dementia with gabapentin or pregabalin use [56], particularly at higher doses. Additionally, Oh et al. associated gabapentin initiation in older adults with functional decline and falls [57]. These mixed findings suggest that while gabapentin may offer benefits for psychosis management in AD, further research is needed to confirm its safety and efficacy in this population.

Our findings suggest the potential benefits of these medications in addressing AD + P, but there are no external studies supporting this conclusion at present. Further clinical trials are needed to validate these results, especially in addressing psychosis in AD patients. More detailed discussion on the specific drug category can be found in the Supplementary Materials.

### 3.3. Specific Protein Roles and Their Mechanistic Impacts in AD + P Patients

In this section, we delve into the important protein targets identified through Method 2 and highlight the drugs that overlap across these targets. These protein targets are associated with multiple drugs and provide insight into the molecular mechanisms that could be leveraged for treatment. Network plot to show the target interaction and following pathway enrichment analysis are in Supplementary Figure S1.

Proteins in the integrin family play a key role in cell adhesion and signaling and may influence the pathological process of AD + P through a variety of mechanisms [58]. For instance, integrin beta-3 (ITGB3) is indirectly linked to AD through its involvement with astrocytes and oligodendrocytes. In the 5xFAD mouse model of AD, ITGB3 expression is increased in reactive astrocytes [59]. Reactive astrocytes are believed to be associated with neuroinflammation and neurodegeneration, which are key features of AD [60]. This point is further supported by a study showing that increased ITGB3 expression in AD mouse colonic tissues is associated with cellular senescence and inflammation. Downregulating ITGB3 was found to reduce these effects, suggesting that ITGB3 contributes to the inflammatory processes in AD [61]. Additionally, Aβ oligomers, a key feature of AD pathology, promote oligodendrocyte differentiation and maturation via ITGB3 and Fyn kinase signaling. These interactions suggest that Aβ influences integrin signaling pathways, impacting oligodendrocyte function and astrocyte reactivity, which are relevant to AD pathogenesis [62]. Integrin alpha-V, as the receptor for irisin on astrocytes, has been proved to facilitate the clearance of Aβ by inducing the release of the Aβ-degrading enzyme neprilysin (NEP) through the downregulation of extracellular singnal regulated kinase – signal transducer and activator of transcription 3 (ERK-STAT3) signaling [63]. Meanwhile, integrin alpha-V has also been reported to mediate synaptic dysfunction in AD by preventing Aβ-induced inhibition of long-term potentiation (LTP) in the dentate gyrus and CA1 regions [64]. Lastly, integrin alpha-L (ITGAL) is primarily known for its role in immune cell trafficking and signaling, particularly in leukocyte adhesion and migration [65]. While it is not as extensively studied in the context of AD as other integrins mentioned here, there is potential for it to be involved in AD due to its role in the immune system. All of this evidence shows that the specified integrins may play significant roles in AD pathogenesis by mediating inflammatory responses, influencing astrocyte and oligodendrocyte functions, and affecting synaptic integrity and Aβ clearance mechanisms.

Calcium channel proteins are essential for neuronal signaling as they regulate calcium ion influx into neurons, affecting neurotransmitter release, synaptic plasticity, and overall neuronal health [20]. The voltage-dependent N-type calcium channel subunit alpha protein is widely expressed in the central and nervous systems and plays a crucial role in neurotransmission both early in development and in the mature peripheral nervous system [21,22,23,24]. Within this family, the voltage-dependent N-type calcium channel subunit alpha-1B (CACNA1B) is linked to AD through its increased expression in response to Aβ exposure. In human SK-N-SH neuroblastoma cells, mRNA levels of alpha-1B, initially lower compared to other subunits, were significantly elevated in a time-dependent manner following Aβ treatment. This suggests that Aβ modulates the transcription of alpha-1B, contributing to disrupted calcium homeostasis and neuronal degeneration in AD [25]. Moreover, calcium channel proteins have also been implicated in the pathology of psychosis. In a study on patients with first episodes of psychosis, it is reported that the patients’ plasma neuron-derived extracellular vesicle levels of calcium channel subunit alpha-1C (CACNA1C) were significantly higher, suggesting its involvement in the altered calcium homeostasis linked to schizophrenia and other neurodevelopmental disorders [26]. Additionally, the CACNA1C risk variant *rs1006737* is associated with reduced white matter integrity in the frontal, parietal, and temporal regions and the cingulate gyrus of individuals with schizophrenia, indicating its impact on brain structure and function in this disorder [27].

Tyrosine hydroxylase, also known as tyrosine 3-monooxygenase, is the enzyme that catalyzes the conversion of the amino acid L-tyrosine to L-3,4-dihydroxyphenylalanine (L-DOPA) [66]. In humans, this enzyme is encoded by the tyrosine hydroxylase (*TH*) gene, and this gene plays a significant role in AD progression and the development of psychosis. Evidence from various studies supports this connection. A meta-analysis found that AD status was associated with decreased dopamine and norepinephrine concentrations, suggesting reduced TH activity [67]. A study using tyrosine hydroxylase heterozygous mutant (*TH*+/−) mice showed that these mice did not develop meth-induced behavioral abnormalities or intracellular signaling changes in the nucleus accumbens, unlike wild-type mice, indicating TH’s role in methamphetamine psychosis [68]. In the context of psychosis in AD, research on spinocerebellar ataxia (SCA) patients with psychosis showed increased *TH* staining in the substantia nigra, indicating a potential link between elevated dopamine production by TH and the development of psychosis [69]. Additionally, a review of various animal models of psychosis showed that dopamine supersensitivity and elevated D2High states, which are often associated with psychosis, are related to increased activity of tyrosine hydroxylase [70]. Interesting, D(4) dopamine receptors, which are targets of anti-Parkinson medications, such as levodopa, ropinirole, and pramipexole, have an RC > 1, indicating an increased risk of AD + P. A more detailed discussion can be found in the Supplementary Materials.

In summary, the protein targets identified in Method 2 offer valuable insights into the molecular mechanisms involved in AD + P. By focusing on key proteins, such as integrins, tyrosine hydroxylase, calcium channels, 3-hydroxy-3-methylglutaryl coenzyme A (HMG-CoA) reductase, and gamma-aminobutyric acid (GABA) receptors, we can begin to uncover the intricate pathways that may contribute to both the onset and progression of psychosis in Alzheimer’s disease. These proteins represent potential therapeutic targets for addressing both cognitive decline and psychotic symptoms, emphasizing the importance of further research into their roles in AD + P pathogenesis and treatment.

### 3.4. Leveraging Data-Driven Models to Identify Beneficial Medications for AD + P Patients

In our study, we leveraged multimodal information, including diagnoses, medication, lab tests, social determinants of health (SDoH), and drug target information, to devise a novel tool for assessing the risk of AD + P. Our recent publication applying the DeepBiomarker framework to predict the adverse outcomes in patients with comorbid post-traumatic stress disorder (PTSD) and alcohol use disorder (AUD) [71] further demonstrates the model’s adaptability and robustness, reinforcing confidence in its utility across diverse neuropsychiatric conditions. AD + P incidence is influenced by various factors, including neurobiology, genetics, psychology, and environmental factors. Integrating real-world data into predictive models is essential for tailoring personalized treatment plans and predicting the occurrence of psychosis. Our primary objective is to develop an algorithm suitable for routine clinical practice while accounting for multiple multimodal factors. The noteworthy strengths of our study include its substantial sample size, convenience, affordability, comprehensive assessments, multidimensional data integration, and real-world applicability. While the identification of optimal biomarker combinations remains an ongoing challenge, our research lays crucial groundwork for identifying beneficial medications and establishing consensus regarding their clinical utility. In this study, we expanded our patient cohort by incorporating data on antipsychotic medications, leveraging a large dataset, and utilizing comprehensive drug target information. We compared our current findings with previous results [18] and identified several new medications (gabapentin, amlodipine, levothyroxine, metoprolol, rivastigmine, statins (simvastatin and rosuvastatin), lisinopril, furosemide, ibuprofen, phenazopyridine, fludrocortisone, glimepiride, sulfamethoxazole, trimethoprim, oxybutynin, and methenamine) while also noting overlap with our previously reported drugs (irbesartan, memantine, vitamin D, esomeprazole, and pantoprazole). This suggests that these drugs consistently interact with the same pathways or targets, reinforcing their significance in the context of AD + P. Additionally, the presence of new medications in our current findings indicates ongoing discoveries and the evolving understanding of drug interactions with their specific protein targets. This approach allowed us to provide a more thorough analysis and refine our understanding of drug–target interactions.

### 3.5. Genetic and Observational Insights into the Link Between Psychosis and Alzheimer’s Disease

Psychosis is a common comorbidity in Alzheimer’s disease (AD + P) and is associated with greater cognitive and functional decline. While our study excluded individuals with pre-existing psychosis before AD diagnosis, genetic and observational evidence suggests a potential link between psychosis and the increased risk of AD. Kodesh et al. reported that very-late-onset schizophrenia-like psychosis (VLOS) was significantly associated with an elevated risk of dementia, including AD, reporting an adjusted hazard ratio of 2.67 [72]. Additionally, GWASs have identified loci associated with psychosis in AD, including *ENPP6* and *SUMF1* [3]. Notably, AD + P demonstrates distinct genetic correlations, including positive associations with depressive symptoms and negative associations with cognitive attainment and bipolar disorder. These findings suggest that psychosis in AD may arise from a unique interplay of genetic and environmental risk factors that warrant further investigation.

### 3.6. Limitation of Our Study

Our study has several limitations. Firstly, while we employed strict inclusion criteria and standardized data inputs to reduce biases inherent in EMRs, challenges, such as selection bias, incomplete data entries, and variability in data quality, may still affect the generalizability of our findings. For example, laboratory tests included in our analysis may not be uniformly performed across all patients, potentially introducing variability. Additionally, some diagnoses and medications might have been underreported, which could influence model predictions. While we explored the influence of biomarkers, patients’ comorbidity situations may have had a more substantial impact, as the diagnostic process incorporates patients’ historical status, whereas biomarkers only reflect their current state. To address these challenges, we plan to expand our dataset in future studies by including records from multiple institutions and diverse patient populations.

Secondly, our study did not consider the direction of target modulations, limiting our understanding of the mechanistic relationships between targets, drugs, and outcomes. Incorporating protein and gene expression data with modulation direction will be a priority in future research to gain deeper insights into these associations.

Additionally, while our study broadly defined psychosis using a comprehensive set of diagnostic codes (Supplementary List S2), future work could focus on stratifying specific psychosis subtypes, such as delusions and hallucinations. This approach could uncover subtype-specific mechanisms and therapeutic responses, advancing precision medicine strategies for managing AD + P.

Moreover, our future research endeavors will encompass the utilization of multiple datasets with other factors (such as stress or social determinants), employing refined algorithms and identifying informative biomarkers. Although external validation on independent datasets was not feasible within the scope of this study, future work will involve validating the model using external datasets, such as Medicare and Optum, to ensure the generalizability of the model. Lastly, while this study identifies potential therapeutic candidates, it does not extend to clinical trials to confirm the efficacy and safety of these medications for AD + P due to the resource availability.

Lastly, while this study identifies potential therapeutic candidates, resource limitations prevented us from conducting clinical trials to confirm their efficacy and safety for AD + P. We hope our findings will inform future preclinical and clinical studies, bridging the gap between computational predictions and real-world applications. Leveraging advanced deep learning models in future work will be critical for analyzing complex datasets and developing more precise predictive models and interventions for managing AD + P.

## 4. Materials and Methods

### 4.1. Data Source

**Figure 2:**
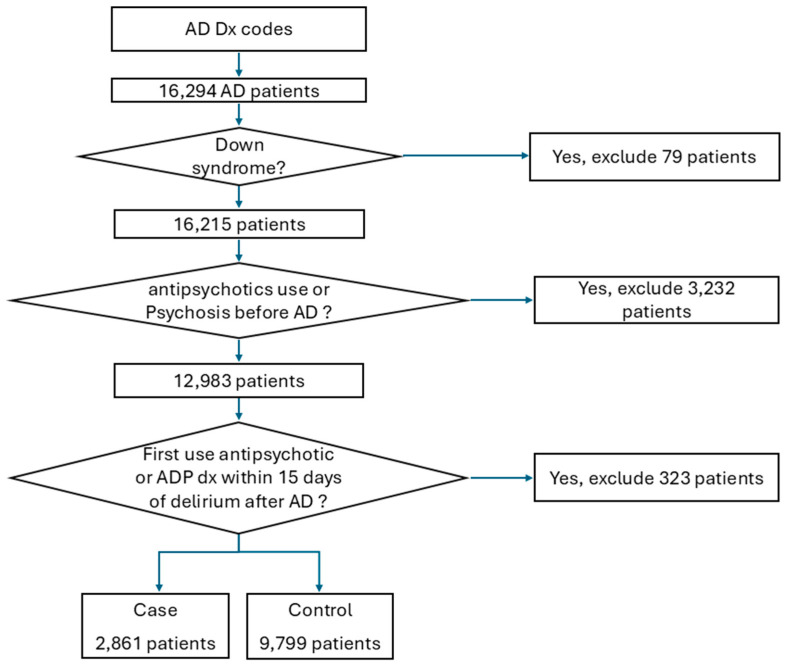
Workflow of inclusion process used in our study.

Data were retrieved from the Neptune system at the University of Pittsburgh Medical Center (UPMC), spanning from January 2004 to October 2019, which manages EMRs within the UPMC health system for research purposes (https://www.rio.pitt.edu/r3-services, accessed on 2 May 2023). The comprehensive database includes a wide range of information, such as demographic details, diagnostic records, medical encounters, prescribed and filled medications, as well as laboratory test results. To identify AD patients and psychosis patients, we utilized specific diagnostic terms within the EMR systems (shown in Supplementary Lists S1 and S2). Additionally, to prevent the potential misclassification of psychosis due to transient delirium symptoms, instances of psychosis diagnosis coinciding with a diagnosis of delirium (outlined in Supplementary List S3) were excluded from the analysis. Workflow of the inclusion process is shown in Figure 2.

### 4.2. Data Preparation

In this study, we aim to predict the likelihood of psychosis development within the next three months for each patient diagnosed with AD based on their EMR history. The inclusion criteria required patients to have (i) a diagnosis of AD, (ii) EMR information available one year prior to the index date, and (iii) no antipsychotic use (order or refill). Additionally, (iv) patients should have had no prior diagnosis of psychosis or antipsychotic use before the index date to ensure that psychosis onset occurs after the AD diagnosis. The primary endpoint of the study is the new onset of psychosis or antipsychotic use (order or refill). Patients meeting these criteria and having a record of psychosis within three months of any encounter are classified as cases, while those with no psychosis record within the same timeframe are classified as controls. If a patient meets the control criteria multiple times, only their latest encounter is considered to reflect their status. Additionally, no psychosis records should exist between this encounter and the index date to confirm a new onset of AD with psychosis. Data augmentation techniques were employed to increase the case number. The index date is defined as the date of the encounter meeting the criteria. Input data included lab test results, diagnoses, and medications from one year prior to the index date. Abnormal lab test results were identified based on specific result flags, and only the top 89 frequently tested lab tests were included. Diagnosis codes were converted from ICD-9 to ICD-10 using a lookup table, resulting in 1614 diagnosis groups. Names of the medications were cross-referenced with DrugBank IDs, producing 1407 unique identifiers. For each clinical encounter, medications, diagnoses, and abnormal lab test results were organized into a sequence, annotated with their respective lab test IDs, diagnosis categories, and DrugBank IDs.

### 4.3. Dataset Splitting

We divided our dataset into three subsets—training, validation, and test sets—using an 8:1:1 ratio.

### 4.4. Drug Target Dataset Collection

While DrugBank provided us with extensive data on drug–target interactions, medications associated with said target, and their therapeutic implications. Leveraging these resources, we built a curated list of AD + P-specific drug targets and drugs. Subsequently, we integrated these into our advanced deep learning model. By incorporating AD + P drug targets as input features into our model, we aimed to enrich the model’s predictive capabilities and enhance its capacity to identify beneficial medications for AD + P. In our previous analysis, we utilized medication histories, diagnostic records, and abnormal lab results to conduct perturbation-based RC analysis, aiming to study the medication impacts. Despite a large sample size involving thousands of cases and controls, the individual sample sizes for specific medications may have been insufficient to reliably detect their effects. To enhance our understanding, our current study employs two innovative approaches: Method 1 (drug-focused analysis) involves perturbation-based importance analysis using drugs, then substituting drugs with their respective targets. These targets are grouped by protein IDs, and their RC is calculated to identify significant drug targets linked to medications. Method 2 (target-focused analysis) shifts focus from drugs to drug targets as inputs, pooling drugs based on shared targets to potentially increase the sample sizes and statistical power. This approach aims to elucidate molecular mechanisms by which medications modulate protein targets and explore medications sharing targets with those in our study population but not directly studied.

### 4.5. The Updated DeepBiomarker Model

**Figure 3:**
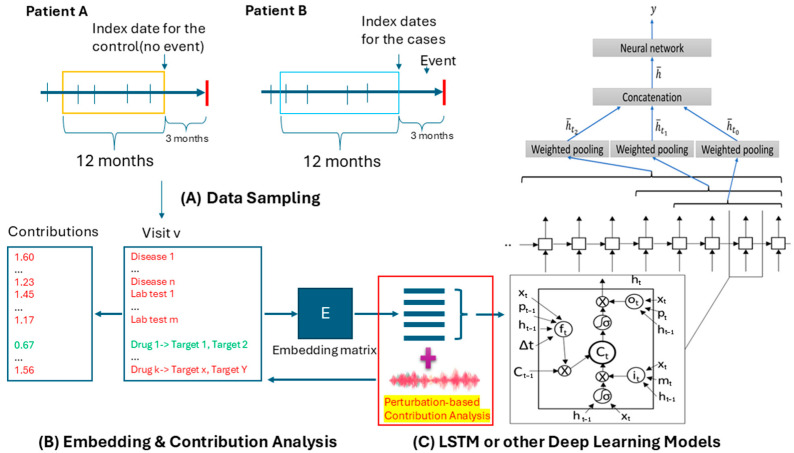
Workflow of updated DeepBiomarker model. (**A**) Data sampling from electronic medical records. Patients meeting the inclusion criteria were categorized based on event occurrence within the specified time interval. Patients without events (Patient A) were classified as controls, while those with events (Patient B) were categorized as cases. Structured EMRs provided multidimensional data inputs, including diagnoses, lab test results, medication usage, and corresponding drug target information. (**B**) Data Embedding. The extracted multimodal data were converted into continuous vector representations to build an embedding matrix. (**C**) Prediction. Neural networks, such as TLSTM and RETAIN, served as the core predictive units in the model. The model produces a comprehensive list of biomarkers, assigning each an RC (relative contribution) value to determine their importance. Biomarkers with RC > 1 indicate high risk, while those with RC < 1 indicate low risk. LSTM: long short-term memory.

As shown in Figure 3, we employed the PyTorch_EHR framework developed by ZhiGroup and applied a range of deep learning models—including recurrent neural networks (RNNs), dilated RNNs (DRNNs), quasi-recurrent neural networks (QRNNs), RETAIN, and T-LSTM—for clinical outcome analyses and predictions. The framework was customized by incorporating data augmentation techniques to improve the model’s performance, integrating individual lab tests and medication data, and adding a contribution analysis module to assess the importance of key factors. In this study, we employed LSTM models, which are characterized by gated memory units to regulate the information flow. These capabilities enable the storage of prior medical history, the inference of the current health situation, and the prediction of relevant clinical outcomes. The parameter settings for the LSTM models in this study were as follows: an embedding dimension of 128, a hidden layer size of 128, a dropout rate of 0.2, two layers, an input size of 30,000, and a patience value of 3. Each deep learning algorithm underwent ten iterations of cross-validation, and the standard deviation of accuracy across these iterations was averaged to measure the accuracy variability in the model’s performance.

### 4.6. Relative Contribution (RC) for Evaluating the Importance of Clinical Factors in the Model

To evaluate the importance of clinical factors in the model’s prediction, we calculated the RC of each feature to the incidence of the outcome [73]. The RC was calculated as the ratio of the median contribution of a feature to events to its median contribution to non-events, with contributions estimated using a perturbation-based analysis approach [74]. The RC value and its statistical significance were computed, as shown below, where FC represents the feature contribution:

$$RC\;value=\frac{{median(FC}_{with\;event})}{{median(FC}_{without\;event})}$$

$$RC\;significance=Wilcoxon\;rank\;sum\;test\;p\;value({FCs}_{with\;event}\;and\;{FCs}_{without\;event})$$

The FC value for a feature was calculated as the sum of its contributions across multiple occurrences within the same patient. In most cases, the FC values did not follow a normal distribution. Therefore, the medians rather than the means of the FC values were used in the RC calculation. The significance of the RCs was determined using a Wilcoxon rank sum test to compare the median FC values between the events and non-events [75]. To mitigate Type I errors from multiple comparisons, we applied the false discovery rate (FDR), representing the expected proportion of false positives among the significant results, with a cutoff value set at 0.05 [76].

To enhance the robustness of our assessment, we normalized the FC values and scaled the RC values across all features. The normalized FC was calculated as the ratio of the contribution of a specific feature to the total contribution of all features. This normalization accounted for variability in the frequency of patient encounters, ensuring that features from patients with frequent visits did not disproportionately influence results. Additionally, since the raw RC values could be affected by imbalances in the visit frequencies between the cases and controls, a scaling factor was applied to ensure balanced and fair evaluations.

### 4.7. Evaluation of the Model’s Performance

The model’s performance was evaluated by the area under the receiver operating characteristic curve (AUROC).

## 5. Conclusions

Our personalized approach has the potential to optimize prevention strategies and alleviate the impact of psychosis in AD patients. Through our findings, we identified several potential medications that may reduce the risk of psychosis among AD patients. While universal prevention programs may provide benefits in the current context, the updated DeepBiomarker model provides more precise and valuable insights. This information can benefit the development of personalized prevention and intervention strategies, which specifically can be tailored to bridge research gaps and address the unique needs of these high-risk patients.

## Figures and Tables

**Table 1:** The performance of the updated DeepBiomarker model with multiple features.

	Validation AUC	Test AUC	Precision	Recall	F1
TLSTM	0.909	0.909	0.851	0.855	0.852
LR	0.797	0.789	0.671	0.822	0.737
RNN	0.728	0.740	0.626	0.890	0.733
DRNN	0.713	0.726	0.597	0.899	0.717
QRNN	0.902	0.904	0.824	0.873	0.847
RETAIN	0.907	0.915	0.900	0.846	0.872
RETAIN (no lab result)	0.904	0.911	0.901	0.842	0.870
RETAIN (no medication)	0.899	0.905	0.888	0.824	0.855
RETAIN (no diagnosis)	0.861	0.858	0.754	0.831	0.789

AUC: area under the curve; TLSTM: time-aware long short-term memory; RETAIN: reverse time attention model; QRNN: quasi-recurrent neural network; LR: logistic regression; RNN: recurrent neural network; DRNN: dilated recurrent neural network.

**Table 2:** Significant medications identified through perturbation-based contribution analysis for predicting psychosis.

Feature Name	Relative Contribution	Wilcoxon *p* Value	FDR-Q *	Bonferroni *p* Value
Phenazopyridine	0.29	3.80 × 10^−10^	8.82 × 10^−8^	6.18 × 10^−7^
Fludrocortisone	0.37	3.52 × 10^−5^	0.001	0.057
Glimepiride	0.39	1.46 × 10^−6^	0.0001	0.002
Irbesartan	0.39	0.00079	0.014	1
Methenamine	0.45	0.0017	0.024	1
Oxybutynin	0.52	1.09 × 10^−8^	1.47 × 10^−6^	1.77 × 10^−5^
Esomeprazole	0.59	0.0019	0.027	1
Sulfamethoxazole	0.6	1.25 × 10^−7^	1.38 × 10^−5^	0.0002
Trimethoprim	0.66	2.48 × 10^−7^	2.24 × 10^−5^	0.0004
Rivastigmine	0.67	2.28 × 10^−7^	2.18 × 10^−5^	0.0004
Ibuprofen	0.67	0.00076	0.013	1
Rosuvastatin	0.68	0.0039	0.049	1
Pantoprazole	0.72	7.91 × 10^−9^	1.29 × 10^−6^	1.29 × 10^−5^
Atenolol	0.76	0.0021	0.029	1
Levothyroxine	0.78	5.82 × 10^−9^	1.05 × 10^−6^	9.46 × 10^−6^
Metoprolol	0.8	1.45 × 10^−6^	0.00011	0.002
Amlodipine	0.82	9.50 × 10^−5^	0.003	0.154
Simvastatin	0.82	0.00016	0.0047	0.263
Furosemide	0.83	0.0037	0.047	1
Lisinopril	0.84	0.00046	0.01	0.752
Donepezil	0.88	0.00013	0.004	0.227
Memantine	0.89	0.0029	0.038	1
Vitamin D3	0.9	0.0034	0.044	1
Ketoconazole	1.96	0.00054	0.011	0.874

* FDR-Q: false discovery rate-adjusted Q-value.

**Table 3:** Significant drug targets and associated medications identified through perturbation-based contribution analysis for predicting psychosis.

Feature Name	Protein Name	Relative Contribution	Wilcoxon *p* Value	FDR-Q *	Bonferroni *p* Value	DrugBank IDs	Drug Names
ITAL_HUMAN	Integrin alpha-L	0.72	7.87 × 10^−10^	6.67 × 10^−8^	1.74 × 10^−6^	DB00095;	Lifitegrast;
						DB00227;	Pitavastatin;
						DB00641;	Rosuvastatin;
						DB01098;	Simvastatin;
						DB08860;	Lovastatin;
						DB11611	Efalizumab
ITB3_HUMAN	Integrin beta-3	0.73	1.11 × 10^−17^	6.11 × 10^−15^	2.45 × 10^−14^	DB00054;	Abciximab;
						DB00063;	Eptifibatide;
						DB00451;	Levothyroxine;
						DB00775;	Tirofiban;
						DB02709;	Resveratrol;
						DB04863	Lefradafiban
ITAV_HUMAN	Integrin alpha-V	0.73	5.97 × 10^−17^	2.37 × 10^−14^	1.32 × 10^−13^	DB00451	Levothyroxine
THA_HUMAN	Tyrosine 3-monooxygenase	0.76	2.31 × 10^−12^	5.10 × 10^−10^	5.10 × 10^−9^	DB00279;	Liothyronine;
						DB00451;	Levothyroxine;
						DB00509;	Dextrothyroxine;
						DB01583;	Liotrix;
						DB04855;	Dronedarone;
						DB05035;	Eprotirome;
						DB01118	Amiodarone
A0A024R8I1_HUMAN	Voltage-dependent N-type calcium channel subunit alpha	0.76	1.51 × 10^−10^	1.58 × 10^−8^	3.32 × 10^−7^	DB00063;	Eptifibatide;
						DB00381;	Amlodipine;
						DB00996	Gabapentin
ATP4A_HUMAN	Potassium-transporting ATPase alpha chain 1	0.78	1.18 × 10^−11^	1.85 × 10^−9^	2.59 × 10^−8^	DB00213;	Pantoprazole;
						DB00338;	Omeprazole;
						DB00448;	Lansoprazole;
						DB00736;	Esomeprazole;
						DB01129;	Rabeprazole;
						DB05351;	Dexlansoprazole;
						DB11739	Vonoprazan
THB_HUMAN	Tyrosine hydroxylase	0.78	1.34 × 10^−10^	1.48 × 10^−8^	2.96 × 10^−7^	DB00451;	Levothyroxine;
						DB00509;	Dextrothyroxine;
						DB03604;	Tiratricol;
						DB05035;	Eprotirome;
						DB01118	Amiodarone
ACE_HUMAN	Angiotensin-converting enzyme	0.78	6.77 × 10^−10^	5.97 × 10^−8^	1.49 × 10^−6^	DB00178;	Enalaprilat;
						DB00492;	Spirapril;
						DB00519;	Cilazapril;
						DB00542;	Captopril;
						DB00584;	Rescinnamine;
						DB00616;	Quinapril;
						DB00691;	Perindopril;
						DB00722;	Lisinopril;
						DB00790;	Moexipril;
						DB00881;	Candoxatril;
						DB01180;	Enalapril;
						DB01197;	Benazepril;
						DB01340;	Trandolapril;
						DB01348;	Fosinopril;
						DB09477	Ramipril
CAC1C_HUMAN	Voltage-dependent L-type calcium channel subunit alpha-1C	0.79	3.43 × 10^−9^	2.61 × 10^−7^	7.56 × 10^−6^	DB00421;	Spironolactone;
						DB01115;	Nifedipine;
						DB01118;	Amiodarone;
						DB00381;	Amlodipine;
						DB01023;	Felodipine;
						DB00343;	Diltiazem;
						DB00153;	Ergocalciferol;
						DB00661;	Verapamil;
						DB00273;	Topiramate;
						DB00243;	Ranolazine;
						DB13961;	Fish oil;
						DB00252;	Phenytoin;
						DB00898;	Ethanol;
						DB00401;	Nisoldipine;
						DB04855;	Dronedarone;
						DB00622;	Nicardipine;
						DB00270;	Isradipine;
						DB00653;	Magnesium sulfate;
						DB00393	Nimodipine
AAKB1_HUMAN	AMP-activated protein kinase catalytic subunit alpha-1	0.80	6.61 × 10^−8^	3.55 × 10^−6^	0.0001	DB00331;	Metformin;
						DB00945;	Aspirin;
						DB12010	Fostamatinib
CAC1B_HUMAN	Voltage-dependent N-type calcium channel subunit alpha-1B	0.82	2.17 × 10^−10^	2.18 × 10^−8^	4.79 × 10^−7^	DB00421;	Spironolactone;
						DB00996;	Gabapentin;
						DB00381;	Amlodipine;
						DB01202;	Levetiracetam;
						DB00153;	Ergocalciferol;
						DB00661;	Verapamil;
						DB00622	Nicardipine
HMDH_HUMAN	3-Hydroxy-3-methylglutaryl-coenzyme A reductase	0.85	2.36 × 10^−7^	1.11 × 10^−5^	0.0005	DB00175;	Pravastatin;
						DB00227;	Lovastatin;
						DB00439;	Cerivastatin;
						DB00641;	Simvastatin;
						DB01076;	Atorvastatin;
						DB01095;	Fluvastatin;
						DB01098;	Rosuvastatin;
						DB04377;	Meglutol;
						DB06693;	Mevastatin;
						DB08860;	Pitavastatin;
						DB09061	Cannabidiol
VDR_HUMAN	Vitamin D3 receptor	0.86	9.70 × 10^−6^	0.00022	0.0214	DB00136;	Calcitriol;
						DB00146;	Calcifediol;
						DB00169;	Vitamin D3;
						DB00910;	Paricalcitol;
						DB01070;	Dihydrotachysterol;
						DB01436;	Alfacalcidol;
						DB02300;	Calcipotriol;
						DB06410;	Doxercalciferol;
						DB11094	Doxercalciferol
GBRA4_HUMAN GBRA6_HUMAN	Gamma-aminobutyric acid receptor subunit alpha-4/6	1.09 1.11	0.0075 0.0047	0.049 0.032	1 1	DB00404;	Alprazolam;
						DB00829;	Diazepam;
						DB00186;	Lorazepam;
						DB00555;	Lamotrigine;
						DB01068;	Clonazepam;
						DB00231;	Temazepam;
						DB00897;	Triazolam;
						DB00241;	Butalbital;
						DB00842;	Oxazepam;
						DB00252;	Phenytoin;
						DB00794;	Primidone;
						DB00898;	Ethanol;
						DB00475;	Chlordiazepoxide;
						DB00628;	Clorazepic acid;
						DB00402;	Eszopiclone;
						DB00690;	Flurazepam;
						DB00683;	Midazolam;
						DB00349;	Clobazam;
						DB01215;	Estazolam;
						DB00418;	Secobarbital;
						DB00371	Meprobamate
FA10_HUMAN	Coagulation factor X	1.26	0.0084	0.047	1	DB01225;	Enoxaparin;
						DB06228;	Rivaroxaban;
						DB13149;	Protein S human;
						DB01109;	Heparin;
						DB00569;	Fondaparinux;
						DB06605;	Apixaban;
						DB09075	Edoxaban
DRD4_HUMAN	D(4) dopamine receptor	1.31	0.0033	0.024	1	DB01235;	Levodopa;
						DB00268;	Ropinirole;
						DB00490;	Buspirone;
						DB00413;	Pramipexole;
						DB01200;	Bromocriptine;
						DB05271;	Rotigotine;
						DB00248;	Cabergoline;
						DB00714;	Apomorphine

* FDR-Q: false discovery rate-adjusted Q-value.

## Data Availability

Data are available upon request from the authors.

## Supplementary Materials

The following supporting information can be downloaded at https://www.mdpi.com/article/10.3390/ijms26041617/s1. References [77,78,79,80,81,82,83,84,85,86,87,88,89,90,91,92,93,94,95,96,97,98,99] are cited in the supplementary materials.
