# Supplementary material for: Exploring Potential Medications for Alzheimer’s Disease with Psychosis by Integrating Drug Target Information into Deep Learning Models: A Data-Driven Approach
Source: Int J Mol Sci. 2025 Feb 14;26(4):1617. doi: 10.3390/ijms26041617 (PMC11855865; doi:10.3390/ijms26041617)
Supplement: Supplementary file 1 [file ijms-26-01617-s001.zip › ijms-3397010-supplementary.pdf]

**Supplementary information:**

**List S1. Diagnosis used for the identification of Alzheimer's disease.**

1.Alzheimer's disease

2.early-onset Alzheimer's disease

3.late-onset Alzheimer's disease

4.Alzheimer's disease, unspecified

**List S2. Diagnosis used for the identification of psychosis.**

1. Unspecified psychosis
2. Senile dementia with delusional features
3. Hallucinations
4. Presenile dementia with delusional features
5. Delusional disorder
6. Depressive type psychosis
7. Other and unspecified reactive psychosis
8. Psychotic disorder with delusions in conditions classified elsewhere
9. Psychotic disorder with hallucinations in conditions classified elsewhere
10. Vascular dementia with delusions
11. Delusional disorders
12. Excitative type psychosis
13. Unspecified psychosis not due to a substance or known physiological condition
14. Hallucinations unspecified
15. Visual hallucinations
16. Psychotic disorder with hallucinations due to known physiological condition
17. Auditory hallucinations
18. Other hallucinations
19. Psychotic disorder with delusions due to known physiological condition
20. Psychogenic paranoid psychosis

**List S3. Diagnosis used for the identification of delirium disorder.**

1.Delirium due to conditions classified elsewhere

2.Vascular dementia with delirium

3.Senile dementia with delirium

4.Subacute delirium

5.Delirium due to known physiological condition

**Supplementary Table S1. Significant diagnoses identified through perturbation-based contribution analysis for predicting psychosis**

| Feature Name | Relative Contribution | Wilcoxon p value | FDR-Q | Bonferroni p value |
|---|---|---|---|---|
| Hyperlipidemia, unspecified | 1.14 | 1.04E-08 | 1.47E-06 | 1.69E-05 |
| Long term (current) use of insulin | 1.16 | 2.79E-10 | 7.56E-08 | 4.54E-07 |
| Unspecified essential hypertension | 1.15 | 1.05E-07 | 1.32E-05 | 0.00017 |
| Esophageal reflux | 1.07 | 0.00045 | 0.0099 | 0.737 |
| Depressive disorder, not elsewhere classified | 1.08 | 0.00061 | 0.0119 | 0.991 |
| Unspecified osteoarthritis, unspecified site | 0.88 | 0.0001 | 0.0033 | 0.163 |
| Type 2 diabetes mellitus without complications | 1.29 | 3.27E-13 | 1.33E-10 | 5.31E-10 |
| Hypothyroidism, unspecified | 1.10 | 0.0016 | 0.024 | 1 |
| Atrial fibrillation | 1.42 | 2.45E-20 | 3.22E-17 | 3.98E-17 |

*FDR-Q: False Discovery Rate-adjusted Q-value

Supplementary Table S1 highlights several common comorbid diseases among AD patients. Several disorders commonly observed in clinical settings, such as hyperlipidemia, hypertension, diabetes, esophageal reflux, atrial fibrillation can be associated with both AD and psychosis patients. Most of those comorbidities require long-term medication use, which could potentially interact with drugs used to manage AD or psychosis symptoms. Therefore, recognizing and managing these comorbidities is essential to optimize treatment outcomes.

**Supplementary Figure S1. A. Target interaction network. B. Pathway enrichment analysis from KEGG**

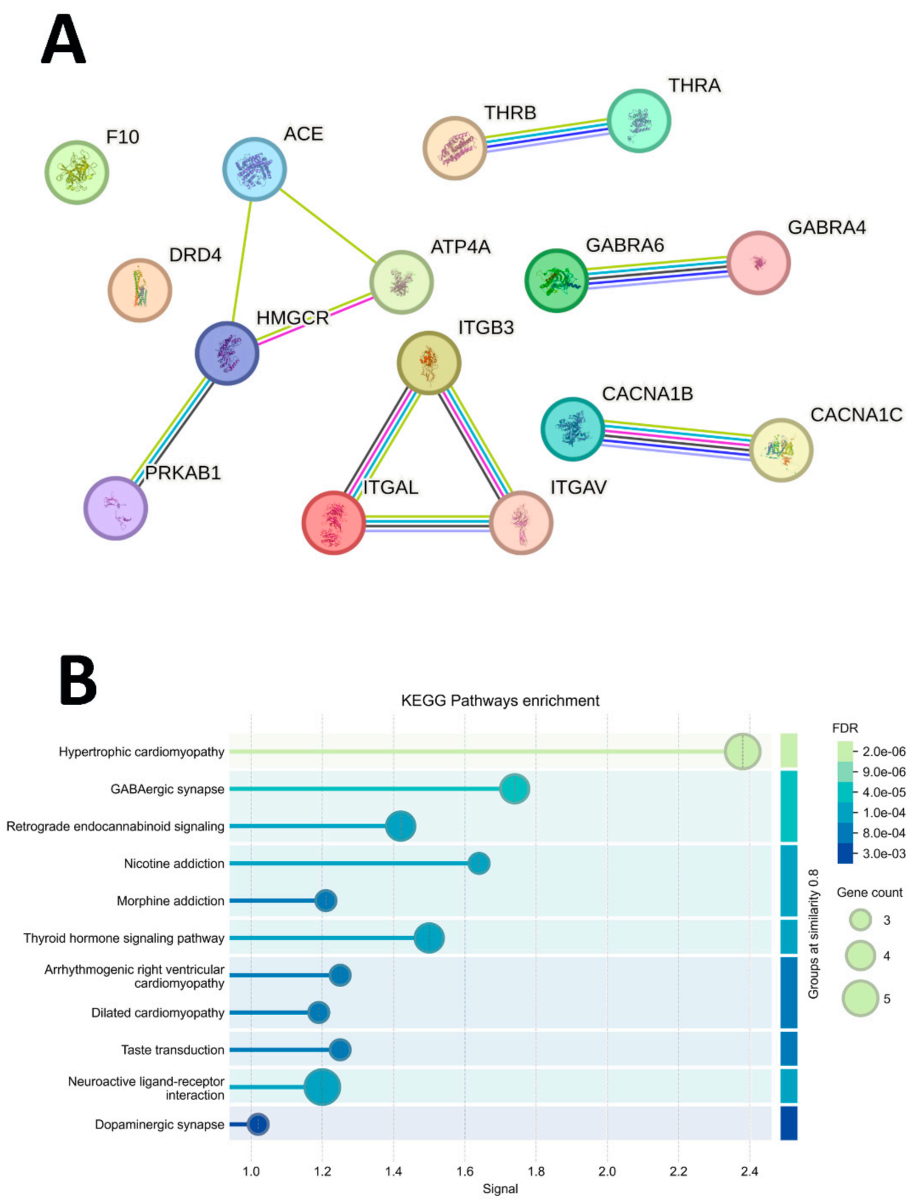


**Detailed Effect of medication use on AD+P prediction**

Statins (Simvastatin and Rosuvastatin): Disruptions in brain cholesterol homeostasis are implicated in AD pathogenesis, suggesting that lipid-lowering medications could influence neurodegenerative processes through cholesterol metabolism or other mechanisms. A longitudinal cohort study, using data from the Swedish Registry for Cognitive/Dementia Disorders linked with national registries, investigated the cognitive impact of lipid-lowering medication use in patients with AD or mixed dementia. They analyzed the mini-mental state

examination (MMSE) scores, the study found that statin use was associated with a dose-response effect: each defined daily dose of statins correlated with an increase of 0.63 MMSE points after 3 years compared to non-use. Simvastatin users showed a significant cognitive benefit over atorvastatin and rosuvastatin users, particularly in younger age groups[1]. Sensitivity analyses focusing on incident users yielded variable results, suggesting the need for further research to confirm these findings in larger, more targeted studies. Additionally, the integrin alpha-V inhibitor Pitavastatin and Simvastatin have been shown to improve psychiatric symptoms, including both negative and positive symptoms, when used as adjunctive therapy[2].

Levothyroxine: Thyroid dysfunction, whether deficiency or excess, can alter mood and cognitive function, leading to anxiety, depression, irritability, and executive function deficits. Hypothyroidism is linked to widespread cognitive decline, particularly memory issues, while thyrotoxicosis primarily affects attention, concentration, and executive functions[3]. AD, the most common form of dementia, is characterized by multidomain cognitive impairment and involves extracellular amyloid deposits and intracellular neurofibrillary tangles. Asher first noted the connection between thyroid dysfunction and cognitive abnormalities in 1949 as "myxoedematous madness," sparking interest in thyroid disorders' role in dementia[4]. Normal brain functions like neurotransmission and memory rely on adequate energy consumption, and low thyroid function can impair cognitive abilities. Studies show thyroid hormones regulate adult brain function, with precise control of thyroid hormone transport, conversion, and receptor levels[5]. Given the risk of cognitive decline with thyroid dysfunction, thyroid diseases may contribute to AD pathophysiology. Clinical and experimental studies suggest a relationship between thyroid hormones and AD, but results are inconsistent due to small sample sizes, participant variability, and limited cognitive test sensitivity[6-9]. Levothyroxine, by normalizing thyroid hormone levels, can potentially improve cognitive function and stabilize mood, reducing AD-related cognitive impairment and psychotic symptoms[10].

Amlodipine: Calcium channel blockers (CCBs) vary in their ability to penetrate the brain. Studies suggest that CCBs may have beneficial effects on psychiatric and neurological disorders, potentially due to their brain penetration[11] . Hypertension is linked to an increased risk of dementia, but studies on antihypertensive treatment and dementia risk have been mixed. Some studies found a reduced incidence of dementia with hypertension treatment, while others did not[12]. Amlodipine has shown potential neuroprotective effects[13]. Another study on hypertensive mice found that amlodipine normalized blood pressure, decreased blood pressure variability, and improved short-term memory, partly by reducing neuroinflammation[14]. In a human study, amlodipine was associated with a lower risk of dementia in patients aged 60 and older[15]. Another study found that in dementia patients, amlodipine reduced mortality and ischemic stroke risk compared to other CCBs, particularly in those with Alzheimer's dementia and dementia with Lewy bodies/Parkinson's disease dementia[16]. These findings suggest that amlodipine may have protective effects against dementia and psychosis in Alzheimer's patients, warranting further investigation into its potential benefits and mechanisms.

Lisinopril: The use of angiotensin-converting enzyme inhibitors (ACEis) has been suggested to alleviate cognitive decline in AD patients, but their protective effects remain debated. Recent studies aimed to evaluate whether oral treatment with the ACEi lisinopril benefits cognitive and physical functions in AD models that overexpresses the human amyloid precursor protein and the human β-site APP-cleaving enzyme in neurons. They observed significant learning, memory, and climbing impairments in young AD flies compared to controls. Additionally, AD flies exhibited a >30-fold increase in the neurotoxic 3-hydroxykynurenine (3-HK) in their heads and higher levels of the reactive oxygen species (ROS) hydrogen peroxide in their muscle-enriched thoraces[17]. Lisinopril treatment significantly improved learning, memory, and climbing abilities in AD flies, likely due to a notable reduction in ROS levels in the thoraces. However, lisinopril did not affect 3-HK levels. These findings provide new insights into the potential therapeutic benefits of ACEis in a preclinical AD model.

Vitamin D: Vitamin D exerts multiple roles in the nervous system, including regulation of neurotrophic factor production, neurotransmitter levels, oxidative stress mechanisms, calcium (Ca2+) homeostasis, and immune system functions, as well as induction of amyloid beta (Aβ) clearance[18]. It has been reported to promote Aβ clearance through enhancing Aβ phagocytosis by macrophages. A study was able to identify potential medications for preventing psychosis in patients with AD. The authors compared medication usage between AD patients with (AD + P) and without (AD - P) psychosis symptoms, and conducted survival analysis to assess time to psychosis associated with different drugs. Molecular mechanisms were explored through gene-signature analysis of drug-induced gene expression profiles, focusing on genes most affected. Vitamin D emerged as significantly more frequently used in AD - P patients and was associated with delayed time to psychosis onset. Genes involved in calcium signaling downstream of the vitamin D receptor were prominently perturbed by vitamin D, suggesting its potential therapeutic role in psychosis prevention in AD[19]. This along with our study highlights vitamin D as a candidate for further drug development efforts targeting psychosis in AD, with genetic variations in vitamin D-regulated genes potentially serving as biomarkers for treatment response.

**Detailed specific protein roles and their mechanistic impacts in AD+P patients**

3-Hydroxy-3-methylglutaryl-coenzyme A reductase, commonly referred to as HMG-CoA reductase, is an enzyme in the mevalonate pathway, which is responsible for the biosynthesis of cholesterol and other isoprenoids[20]. It catalyzes the conversion of HMG-CoA to mevalonate, a key regulatory step in the production of cholesterol[20]. Interestingly, PRKAA1 and HMGCR are connected through the regulatory role of AMPK over HMGCR, which shows the potential benefits of synergistic therapy targets in managing the AD + P. What is more, a pharmacogenetic study already proved that HMGCR variants will impact the anxiety and depression situation in AD[21], which makes our predicted target, HMGCR, more promising in the AD + P prediction.

Gamma-aminobutyric acid receptor subunit alpha-4/6 are component protein of Gamma-aminobutyric acid (GABA), whose major role in human brain is inhibitory neurotransmitter[22]. Their neurofunction could not be discussed with its important precursor – glutamate. They are

targets of benzodiazepines which are used for the treatment of anxiety. And benzodiazepines act as positive allosteric modulators. A review paper indicates that in psychosis, the positive correlations between anterior cingulate cortex (ACC) glutamate levels and brain activity are diminished during resting states but are enhanced during tasks requiring cognitive control[23]. There is also evidence suggesting that antipsychotic medications can modify the relationship between glutamate levels and brain activity[23].


1. Petek B HH, Xu H, et al. Statins and cognitive decline in patients with Alzheimer's and mixed dementia: a longitudinal registry-based cohort study. Alzheimers Res Ther. 2023;15(1):220. Published 2023 Dec 20. doi:10.1186/s13195-023-01360-0.
2. Shen H LR, Yan R, et al. Adjunctive therapy with statins in schizophrenia patients: A meta-analysis and implications. Psychiatry Res. 2018;262:84-93. doi:10.1016/j.psychres.2018.02.018.
3. Kim JH LH, Kim YH, et al. The Association Between Thyroid Diseases and Alzheimer's Disease in a National Health Screening Cohort in Korea. Front Endocrinol (Lausanne). 2022;13:815063. Published 2022 Mar 7. doi:10.3389/fendo.2022.815063.
4. Asher R. Myxoedematous madness, Br Med J 1949;2:555-562.
5. Schroeder AC PMTh, t3 and t4, in the brain. Front Endocrinol (Lausanne). 2014;5:40. Published 2014 Mar 31. doi:10.3389/fendo.2014.00040.
6. de Jong FJ dHT, Visser TJ, de Rijke YB, Drexhage HA, Hofman A, et al.. Thyroid Hormones, Dementia, and Atrophy of the Medial Temporal Lobe. J Clin Endocrinol Metab (2006) 91:2569–73. doi: 10.1210/jc.2006-0449.
7. Luo L YN, Mao Q, Jackson I, Stopa E. Thyrotropin Releasing Hormone (TRH) in the Hippocampus of Alzheimer Patients. J Alzheimers Dis (2002) 4:97–103. doi: 10.3233/JAD-2002-4204
8. O'Barr SA OJ, Ma C, Brent GA, Schultz JJ. Thyroid Hormone Regulates Endogenous Amyloid-β Precursor Protein Gene Expression and Processing in Both In Vitro and In Vivo Models. Thyroid (2006) 16:1207–13. doi: 10.1089/thy.2006.16.1207.
9. Van Osch LA HE, Combrinck M, Smith AD. Low Thyroid-Stimulating Hormone as an Independent Risk Factor for Alzheimer Disease. Neurology (2004) 62:1967–71. doi: 10.1212/01.WNL.0000128134.84230.9F.
10. Uma D RR, Lee JH, Gavini DR, Shah PH, Hamid P. Does Hormone Supplementation With Levothyroxine Improve Hypothyroid Impaired Cognitive Dysfunction?. Cureus. 2021;13(9):e17885. Published 2021 Sep 11. doi:10.7759/cureus.17885.
11. Colbourne L, Harrison PJ. Brain-penetrant calcium channel blockers are associated with a reduced incidence of neuropsychiatric disorders, Mol Psychiatry 2022;27:3904-3912.

12. Lennon MJ LB, Lipnicki DM, et al. Use of Antihypertensives, Blood Pressure, and Estimated Risk of Dementia in Late Life: An Individual Participant Data Meta-Analysis. JAMA Netw Open. 2023;6(9):e2333353. doi:10.1001/jamanetworkopen.2023.33353.
13. Park H, Han, MH., Choi, H. et al. Mitochondria damaged by Oxygen Glucose Deprivation can be Restored through Activation of the PI3K/Akt Pathway and Inhibition of Calcium Influx by Amlodipine Camsylate. Sci Rep 9, 15717 (2019). https://doi.org/10.1038/s41598-019-52083-y.
14. Kerkhofs D HR, Hermes D, et al. Amlodipine limits microglia activation and cognitive dysfunction in aged hypertensive mice. J Hypertens. 2023;41(7):1159-1167. doi:10.1097/HJH.0000000000003445.
15. Feldman L VS, Efrati S, et al. Amlodipine treatment of hypertension associates with a decreased dementia risk. Clin Exp Hypertens. 2016;38(6):545-549. doi:10.3109/10641963.2016.1174249.
16. Kalar I XH, Secnik J, et al. Calcium channel blockers, survival and ischaemic stroke in patients with dementia: a Swedish registry study. J Intern Med. 2021;289(4):508-522. doi:10.1111/joim.13170.
17. Thomas J SH, Smith CA, et al. The Angiotensin-Converting Enzyme Inhibitor Lisinopril Mitigates Memory and Motor Deficits in a Drosophila Model of Alzheimer's Disease. Pathophysiology. 2021;28(2):307-319. Published 2021 Jun 18. doi:10.3390/pathophysiology28020020.
18. Landel V AC MP, Morello M, Féron F. Vitamin D, Cognition and Alzheimer's Disease: The Therapeutic Benefit is in the D-Tails. J Alzheimers Dis. 2016;53(2):419-444. doi:10.3233/JAD-150943.
19. Wang L YJ FP, et al. Effects of Vitamin D Use on Outcomes of Psychotic Symptoms in Alzheimer Disease Patients. Am J Geriatr Psychiatry. 2019;27(9):908-917. doi:10.1016/j.jagp.2019.03.016.
20. Friesen JA RVT-h--mc-AH-CrGBdg----.
21. Cacabelos R CJ, Corzo L, et al. Pharmacogenetics of anxiety and depression in Alzheimer's disease. Pharmacogenomics. 2023;24(1):27-57. doi:10.2217/pgs-2022-0137.
22. Jewett BE SSP, GABA. [Updated 2023 Jul 24]. In: StatPearls [Internet]. Treasure Island (FL): StatPearls Publishing; 2024 Jan-. Available from: https://www.ncbi.nlm.nih.gov/books/NBK513311/.
23. Zahid U OE, Hedges EP, et al. Neurofunctional correlates of glutamate and GABA imbalance in psychosis: A systematic review. Neurosci Biobehav Rev. 2023;144:105010. doi:10.1016/j.neubiorev.2022.105010.
